# Investigating the Usability, Efficacy and Accuracy of a Medication Entering Software System for a Healthcare Robot

**DOI:** 10.3389/frobt.2022.814268

**Published:** 2022-01-25

**Authors:** Nataly Martini, Elizabeth Broadbent, Jasmine Koo, Laurence Lam, Diane Verches, Sophie Zeng, Rhea Montgomery-Walsh, Craig Sutherland

**Affiliations:** ^1^ School of Pharmacy, Faculty of Medical and Health Sciences, University of Auckland, Auckland, New Zealand; ^2^ School of Psychological Medicine, Faculty of Medical and Health Sciences, University of Auckland, Auckland, New Zealand; ^3^ Department of Electrical, Computer and Software Engineering, Faculty of Engineering, University of Auckland, Auckland, New Zealand

**Keywords:** health information systems, pharmacists, medication entering software system, human-computer interaction, healthcare robot

## Abstract

**Purpose:** This research aimed to evaluate medication software for a healthcare robot. Study I compared two software versions (RoboGen and RoboGen2) for system usability, speed and accuracy of medication entry; Study II evaluated system usability and community pharmacists’ views of RoboGen2.

**Methods:** Study I had a within-subjects experimental design and recruited 40 Health Sciences students to enter different, comparable sets of prescriptions into the two systems, in randomized order, within a limit of 15 min. Screen activity was recorded to observe prescription errors. Study II had a cross-sectional observational design and recruited 20 community pharmacists using convenience sampling. Pharmacists entered three prescriptions using RoboGen2. Participants in both studies completed the System Usability Scale (SUS) following each task. Study I participants completed a questionnaire on system preference, and Study II participants a semi-structured interview.

**Results:** Study I participants preferred Robogen2 (*p* < 0.001) due to its sleek and modern layout, good flow, ease of use, and intuitive design. SUS scores [*t* (40) = −3.40, *p* = 0.002] and speed of medication entry favored Robogen2 (*t* = 3.65, *p* < 0.001). No significance was found in accuracy (*t* = 1.12, *p* = 0.27). In study 2, pharmacists rated the usability of RoboGen2 below average. Themes from interviews were navigation and streamlining the system, ease of use, and integration with pharmacy software systems.

**Conclusion:** Adding safety features and better aesthetics can improve the usability and safety of a medication prescription system. Streamlining workflow and pre-populating data can increase speed of prescription entry without compromising patient safety. However, a better approach is integration with pre-existing pharmacy systems to reduce workload while incorporating safety features built into existing dispensing systems.

## 1 Introduction

Chronic health conditions affect nearly one in four New Zealand (NZ) adults (Ministry of Health, 2020a) and contribute towards almost 90% of healthy life lost due to early illness, disability, or death (Ministry of Health, 2020b). The World Health Organization predicts that globally, chronic diseases account for 41 million deaths each year (World Health Organization, 2021). Whilst significant advances in healthcare have seen a substantial decline in mortality, this has also meant an increase in multiple long-term medications. With greater medication regimen complexity comes a greater risk for reduced medication adherence (Rantanen et al., 2017). Evidence suggests that adherence to long-term therapy is suboptimal with almost 40% of people stopping their medicines after the first year of therapy (Torres-Robles et al., 2021). Factors contributing to poor medication adherence include intentional factors, where patients deliberately forego treatment, or unintentional reasons such as forgetfulness (Sabaté and World Health Organization, 2003). As people age and/or take more medicines, forgetfulness becomes more common (Patton et al., 2017).

Medical device solutions designed to target forgetfulness include memory aids such as reminder packaging, text messaging, automatic prescription refills, and electronic reminders and monitoring devices (Rantanen et al., 2017). Alarm-based aids and pill-monitoring devices are active solutions that, when connected to the internet, can inform the patient if a dose is missed. Research has shown these aids act most effectively when combined with assistance from a care provider (Granger and Bosworth, 2011; Velligan et al., 2013). Other solutions include software-based systems, such as personal health portals and interactive/social robots, where users can share health-related information and receive personalized feedback (Datta et al., 2011). Social robots are emerging technologies that show promise for improving medication adherence by acting as medication reminders (Broadbent et al., 2018), as well as serving as social companions and coordinating patient medication information with healthcare professionals and/or carers (Rantanen et al., 2017). However, for health technologies to be usable, they need to be well designed, intuitive, easy to use, and meet the profession’s standards (Johnson et al., 2005). Failing to incorporate these features effectively may lead to inefficient care and prove time-consuming and/or labor-intensive (Mansoor et al., 2014; Hall et al., 2016; MacLure and Stewart, 2016; Pinto et al., 2018; Navti and Apampa, 2019). Furthermore, the aesthetics of an application can influence perceived usability even if there are no differences in functionality offered (Kurosu and Kashimura, 1995; Tractinsky, 1997).

**FIGURE 1:**
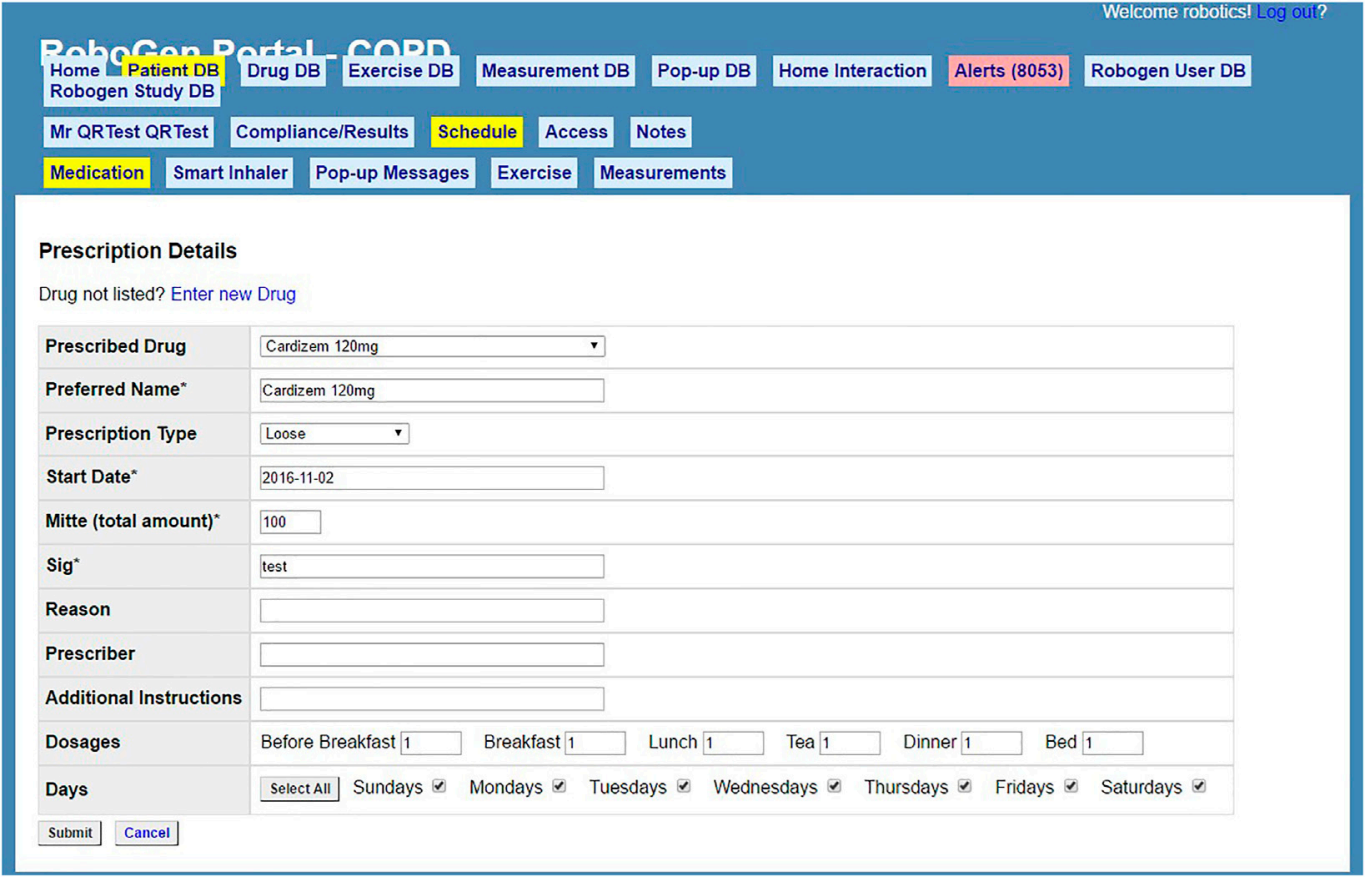
RoboGen user interface: medication selection.

In 2011, RoboGen, a web-based application, with enabled end-user programming, was designed by a team of health informatics and electrical and computer engineering researchers at the University of Auckland (Figure 1) (Datta et al., 2011). RoboGen was designed to be used by healthcare professionals as a medication management support system on a healthcare/social robot for residents in a retirement village (Datta et al., 2011, 2012; Broadbent et al., 2014). Field studies suggested that a robotic platform offered opportunities that were not possible with other medication management systems, and end-user programming allowed health professionals to manage medication instructions, dosing schedules, health education, appointment and refill reminders (Datta et al., 2011). Subsequently, RoboGen was expanded to specifically support patients with chronic obstructive pulmonary disease (COPD) by providing patient education, and in managing medication adherence, rehabilitation exercises, monitoring symptoms, and peak expiratory flow (Broadbent et al., 2018). During the COPD study, issues were reported by healthcare professionals entering patients’ prescriptions, in particular, that medication names and doses had to be typed in and all entries were saved in the system, resulting in a confusing collection of different spellings, generic names, and dosages.

**FIGURE 2:**
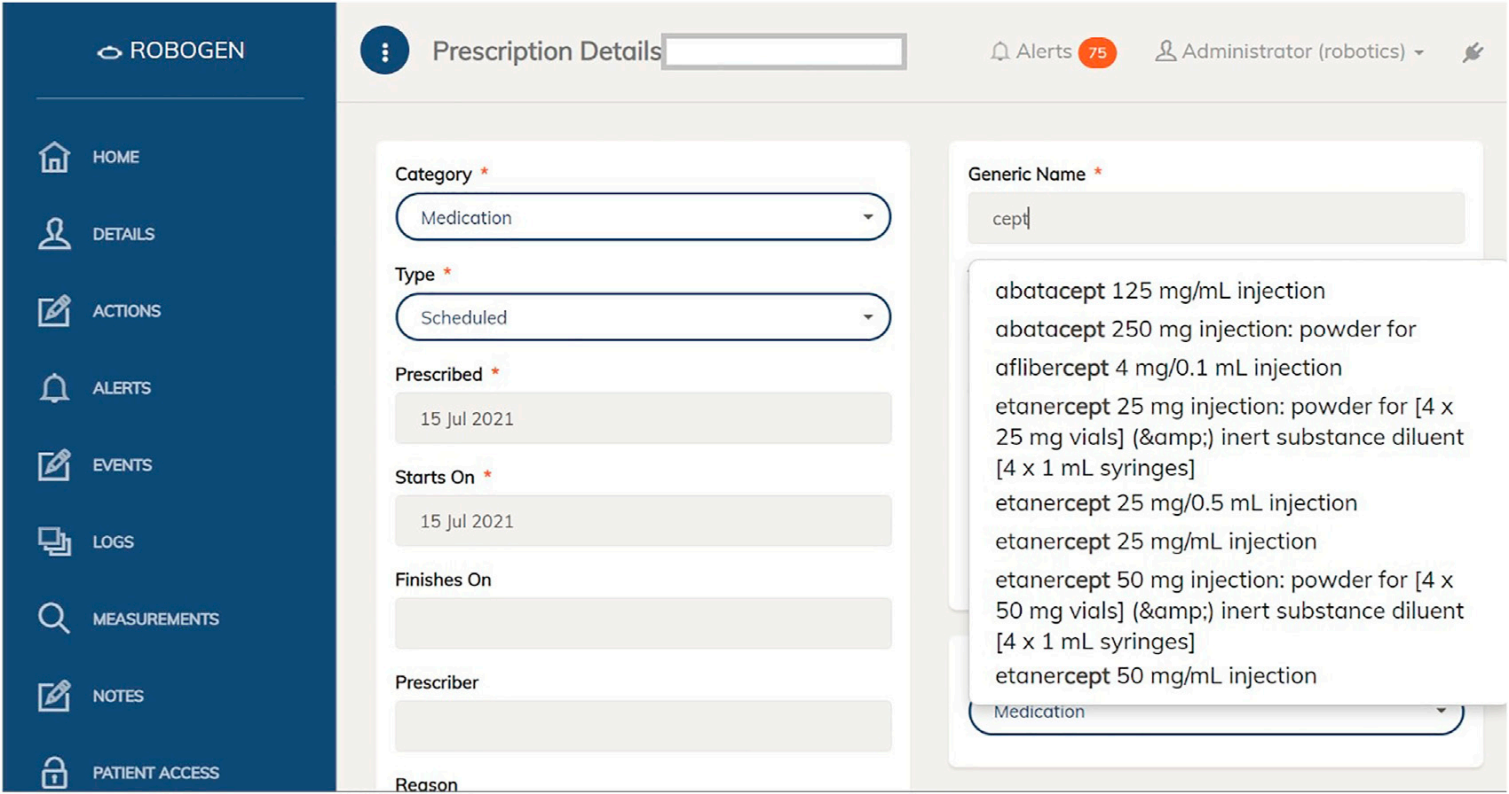
RoboGen2 user interface: medication selection search function.

**FIGURE 3:**
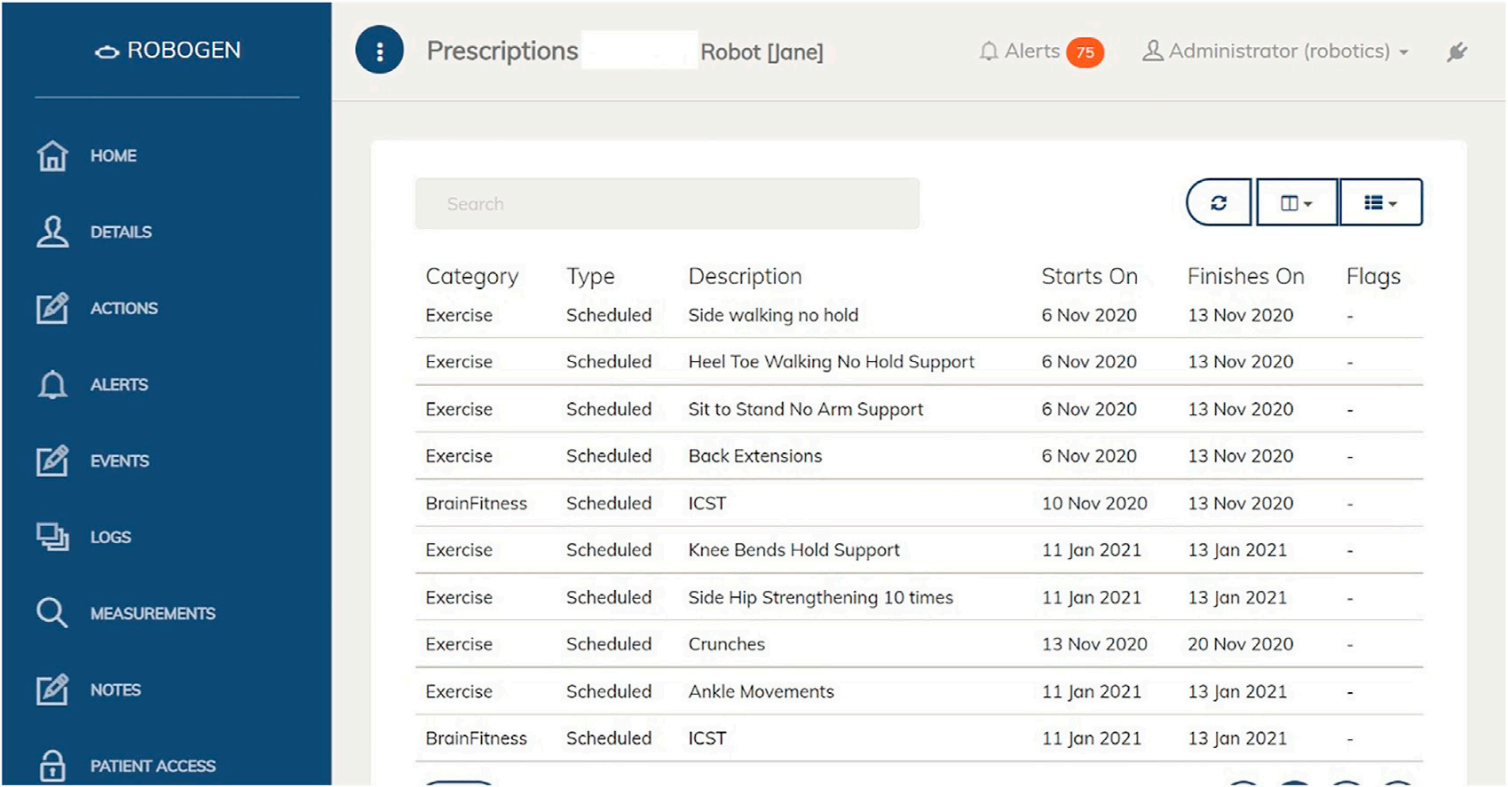
RoboGen2 test patient exercise information.

The overall research problem that this study aimed to address was how to make the medication management software more usable for healthcare professionals. In response, RoboGen2 was developed to include a different user interface, an imported list of medications consistent with the New Zealand Universal List of Medications (NZULM), and supplementary safety features (Figure 2 and Figure 3). An official drop-down list of medications and doses was used instead of typing names and doses.

To determine whether these changes were an improvement on the original RoboGen software and were better able to meet the requirements of healthcare professionals entering medication on healthcare robots, the following research questions were proposed:

1) What is the difference in system usability, efficiency and accuracy of medication entry between RoboGen and RoboGen2? The authors hypothesized that RoboGen2 would be superior to RoboGen in system usability, efficiency and accuracy.
2) How usable do pharmacists find RoboGen2, and what are their views on using the software to support patient and caregivers to monitor medication adherence?

To answer the research questions and test the hypothesis, the research was designed as two studies. Study I aimed to compare the two versions of the medication entering software systems (RoboGen with RoboGen2) with participants who had some familiarity with medication entry to determine whether the changes to RoboGen system affected the usability, speed, and error rate of medication entry. Study II aimed to test system usability of RoboGen2 with pharmacists, who are licensed to dispense prescription medicines and use other medication management systems in the workplace, and to gather their views on using the software. Few studies on medication management software have involved pharmacists.

## 2 Materials and Methods

Studies I and II had approval from the University of Auckland Human Participants Ethics Committee (Reference numbers 020792 and 021323).

### 2.1 Study I

Study I was an experiment with a within-subjects design. Students enrolled in Health Sciences disciplines were recruited from the Faculty of Medical and Health Sciences at the University of Auckland via email, lecture announcements, and posters around campus. Inclusion criteria were students over the age of 16, familiarity with medication and/or prescriptions entry, and English language competence. After providing consent, participants completed a baseline demographic questionnaire, were given a demonstration of a prescription entered into each system and were asked to perform the same task with both RoboGen and RoboGen2. The task required participants to enter a set of fictional prescriptions, provided by the School of Pharmacy, into the system as accurately and efficiently as possible, with an imposed time limit of 15 min, to reflect a realistic pharmacy practice. Participants entered one set of prescriptions into the original RoboGen, and a different, comparable set into RoboGen2. The order of items in each of these sets of prescriptions was randomized as was the order of versions used by each participant. The study was conducted July–August 2018.

The independent variable was the version of RoboGen, and the dependent variables were the speed and accuracy with which the prescriptions were entered, self-reported usability, and preference. Following each task, participants were asked to complete the System Usability Scale (SUS) (Bangor, 2009) scored out of 50, with reliability analysis, Cronbach’s alpha of 0.91. The questionnaires included open-ended questions about likes and dislikes of each system, and which system participants preferred. Screen activity was recorded to observe prescription errors. Data were analyzed using a chi-square goodness-of-fit test for system preference, and paired sample *t*-tests to compare the two systems in terms of usability, speed (number of prescriptions entered in 15 min), and the difference in the proportion of correct entries (i.e., accuracy) between the two systems. Initial results from Study I on usability have been published in a brief report which did not include accuracy or speed results (Broadbent et al., 2020).

### 2.2 Study II

This was a cross-sectional observational study that included both a survey and interview. Pharmacies were recruited within central Auckland using convenience sampling, and data were collected from June–September 2018. Email addresses and phone numbers of pharmacies were obtained from the online portal Healthpoint. co.nz. Email invitations were sent to managers of the first 100 pharmacies together with a participant information sheet and consent form. Researchers followed up with a phone call 1 week after sending the email to enquire whether pharmacists wished to participate. A time and place for where the study would occur was negotiated with the 20 pharmacists expressing interest in participating. Recruitment of multiple pharmacists from the same pharmacy was allowed.

After consenting, pharmacists were asked to complete a short demographic questionnaire, and enter three fictional prescriptions using RoboGen2, each containing oral medications with various dosing instructions. Subsequently, pharmacists were invited to complete the SUS, and participate in an interview. Semi-structured interview questions were developed to enable open-ended opinions of the RoboGen2 software, as well as questions related to technology used in pharmacy, reported elsewhere (Law et al., 2021). The interview was recorded with a digital voice recorder and transcribed verbatim. Transcribed interviews were checked for accuracy by sending these to participants who wished to review these before analysis, and entered into NVivo 12 (QSR International Pty Ltd. Version 12, 2018). An inductive approach was used to thematically analyze and code the qualitative data (Thomas, 2006). Researchers individually read through the transcripts to familiarise themselves with the data and grouped them into common ideas or patterns (codes). Through a series of group meetings, codes were discussed and collectively developed as themes.

## 3 Results

### 3.1 Study I—Health Sciences Students

Participants’ age ranged from 18 to 48 years with the majority of the 40 participants recruited to the study identifying as female (*n* = 27), Asian (*n* = 20), having moderate (*n* = 18) data entry experience, and representing undergraduate pharmacy (*n* = 12) (Table 1).

**TABLE 1:** Study I participant demographics (N = 40).

		n (%)
Age (mean)	25.2 (SD 7.15)	
Sex	Female	27 (67.5)
	Male	13 (32.5)
Ethnicity	Asian	20 (50)
	European/NZ European	14 (35)
	Māori/Pacific	4 (10)
	Other	2 (5)
Undergraduate students	Pharmacy	12 (30)
	Medicine	9 (22.5)
	Nursing	3 (7.5)
	Optometry	2 (5)
	Other	5 (12.5)
Postgraduate students	PhD	7 (17.5)
	Masters	2 (5)
Data entry experience	Very experienced	3 (7.5)
	Moderate experience	18 (45)
	Little experience	15 (37.5)
	None	4 (10)

**FIGURE 4:**
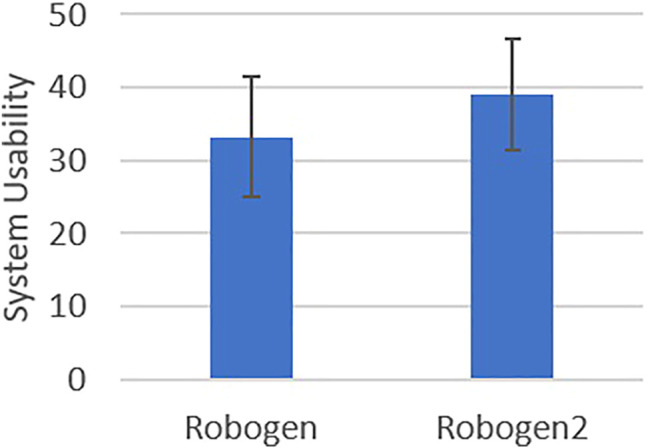
System usability of RoboGen versus RoboGen2.

**FIGURE 5:**
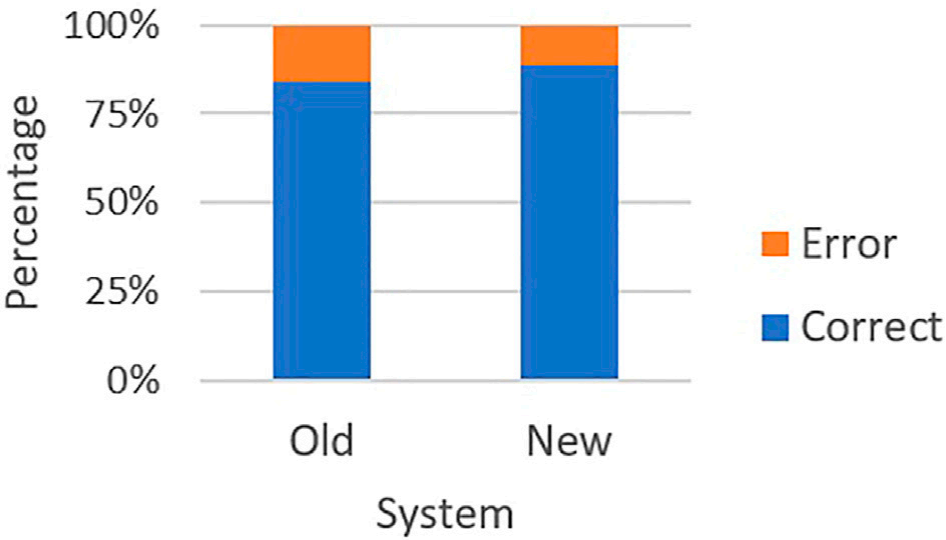
Errors by system. The red bars indicate errors and the blue bars indicate correct entries.

The mean usability scores for the two systems were significantly different {RoboGen = 33.27, SD 8.31, RoboGen2 = 39.12, SD 7.66), *t* (40) = −3.40, *p* = 0.002, mean difference −5.85 (95% CI [−9.12, −2.57]} (Figure 4). The speed of prescription entry within the allocated time was significantly faster for RoboGen2 compared with RoboGen [*t* (40) = 3.65, *p* < 0.001], with a mean difference of 1.87 min (95% CI [0.83, 2.91]). Accuracy of prescription entry error by system showed there was no significant difference in the proportion of correct prescription entries between the two systems (*t* = 1.12, *p* = 0.27), with the mean difference 0.04 (95% CI [-0.29,0.75]) (Figure 5).

The main errors for RoboGen were incorrect medication name (including spelling mistakes) and incorrect number of medicines provided, e.g., converting 1/12 (1-month supply) to 1 instead of 30. Incorrect number of medicines provided was also common for RoboGen2, although this was more likely to be typed incorrectly instead of a conversion error. Another common error was selecting an incorrect generic name when converting from a trade (brand) name (e.g., “levonorgestrel 1.5 mg tablet” instead of “levonorgestrel 150 μg + ethinylestradiol 30 μg tablet” for Ava 30 ED tablets). Both systems had an equal number of errors with transposing first/last patient names or spelling errors.

Significantly more participants (*n* = 32) preferred RoboGen2 to Robogen 1 (*n* = 8) [Chi-Square (1,14) = 14.40, *p* < 0.001]. Of the 12 pharmacy students, seven preferred Robogen2 and five preferred Robogen. The majority of participants preferred RoboGen2 due to its sleek and modern layout, good flow, ease of use, and intuitive design. Participants perceived it to be more efficient (hence less time-consuming) and selecting medicines from a dropdown list was believed to allow for more accuracy, reducing risk of dispensing error. Some participants thought this system would better support medication entry for those with less medicine knowledge. Limitations of the system included the listing of medicines by generic name only. This made finding the trade name difficult, particularly for medicines with multiple active ingredients such as oral contraceptives. Some participants wanted more options to enter patient data and autofill options for medication dosing instructions. Other restrictions were using a mouse to navigate rather than arrow keys, the inability to delete a prescription, a small dropdown box for medicines, and need for multiple clicks to navigate between screens.

Those who preferred RoboGen thought it was a simpler interface allowing users to input additional patient information, and manual medicine entry was considered easier than sifting through a dropdown list. Previously entered medicines were saved, which meant they appeared in future searches and allowed for faster entry as more prescriptions were added, saving time with repeat prescriptions. Limitations were the user interface, which was seen to be “ugly,” outdated, and visually confusing. Participants thought it presented too many options, wasn’t intuitive, didn’t offer auto-fill options and the navigation was puzzling. Some participants felt it was a “real brain drain” staying focussed while using this system.

### 3.2 Study II—Community Pharmacists

Twenty pharmacists were recruited of which the majority were female (*n* = 11) and aged between 20 and 30 years (*n* = 13) (Table 2).

**TABLE 2:** Study 2 participant demographics (N = 20).

		n (%)
Age	20–30	13 (65)
	31–40	3 (15)
	41–50	1 (5)
	51–60	2 (10)
	>60	1 (5)
Sex	Female	11 (55)
	Male	9 (45)

The overall SUS score was 67.7, a marginal C-grade. According to the survey, most pharmacists found the task easy to do and were confident in performing the task, but were ambivalent as to whether they would like to use the system frequently.

Three main themes were developed from the interviews, namely navigation and streamlining the system, ease of use, and integration with pharmacy software systems (selected quotes in Table 3).

**TABLE 3:** Selection of community pharmacists’ quotes.

Theme	Quote
Navigation and streamlining the system	“Software needs to flow a little better. For example if you had the patient’s information and what medicines they were prescribed, you could still see their details, the prescriber details at the top, that would be awesome. And then you could have medicines underneath them I guess. Definitely flow better, don’t know why I had to go back at one point. Why couldn’t it go ‘next’, I guess when I had a new medicine.”—P20
	“I’d rather have something going “next, next” rather than going back. Because that made me think twice, am I going back a page or going back? So if it’s me I would say enter patient details, “next”, enter patient’s medicines, “next”, add another medicine, something along those lines rather than going back. Because when there’s too many tabs that can cause confusion, like I can interpret it differently to another person to how they would interpret it, but then if there’s just a next button you can keep going from one step to another and go back in and edit something and then next.—P11
Ease of use	“Because it’s quite easy to use, but there’s a lot of tabs. So if there were just three or four tabs, would make it way easier. But once you get to know the tabs, it’s probably not so bad. But there must be a way that you don’t have to go back, and then enter, and then go back. Which would save a bit of time.”—P10
	“As a pharmacist I don’t want to be using a software and having to put a new medicine each time. It’s quite a big barrier and slows me down and one big thing in pharmacy is time. You wanna make it so it’s extremely easy. You don’t want somebody hunting around for things … you should just be able to go next medicine and whatnot and just add them very fluidly.”—P19
	“… not having to use mouse like you do with Toniq you could just use a keyboard basically and it saves a lot of time … And muscle memory kinda takes over and you can just do it so much faster.” P4
Integration with pharmacy software systems	“If you are inputting it into TONIQ and then inputting it into the RoboGen software, it’s double the work and a pharmacist won’t do it. Yeah, probably just a bit more integration.”—P7
	“I think it will have to be tied in with our current software provider. Tie it all in with the software provider so it is all up to date and current with the medicines. I wouldn’t want to enter in every new medicine myself. And its only as good as the person that enters them … It’s got what it needs in there and like everything you enter it in once and it’s all good so I think that’s fine.”—P13
	“Well the main thing is to link it up to the dispensing programmes that you already use. Yeah, so we don’t have to do it again. Or like the interactions and stuff … like in the drug database things can be uploaded and connected to like NZF then that would pop up with the interactions as well ‘cause we’ll have to keep looking back at the references.”—P5
	“I basically found it a bit of a nuisance because as I was putting the drugs in, I had to constantly go to another computer to check whether it’s got to be taken with food and alcohol and all interactions and all that. Whereas our software is already doing all that. So yeah, that was a problem. It’s just doubling error, you know there’s an error possibly of me typing things here and typing things there. Putting the wrong name in, maybe the wrong date of birth.” - P3

#### 3.2.1 Navigation and Streamlining the System

Although most pharmacists were able to navigate the system fairly easily, some did express that the software was “a bit clunky and difficult to navigate” initially. Several pharmacists thought that the process of entering prescription details could be designed to “flow better.”

#### 3.2.2 Ease of Use

While most pharmacists found the software relatively straightforward to use, some believed that re-entering medicines with each new prescription was time-consuming and awkward. A few pharmacists suggested that if the software was connected to the medication database as their current dispensing software was, it would make the process much faster and hence easier to use. Others suggested that using a keyboard would be a faster way to enter information, which is what they were used to with their pharmacy dispensing databases.

#### 3.2.3 Integration With Pharmacy Software Systems

The majority of pharmacists believed that integration with their current pharmacy dispensing software (Toniq™ or LOTS™) was not only a way to be more time-efficient, but also ensure patient medicines were up to date. Furthermore, their current dispensing programs had inbuilt drug interaction checking features and were connected to drug formularies where pharmacists could check for drug information. They believed that having another system would potentially increase the risk of dispensing errors.

## 4 Discussion

This study demonstrates that Health Sciences students preferred RoboGen2, which resulted in significantly higher usability scores and speed of medication entry when compared with an older version, but with no significant differences in the accuracy. Interviews with community pharmacists suggest that the usability of RoboGen2 could translate well into their practice, but substantial changes would need to be made to better integrate healthcare robot software into existing pharmacy dispensing systems.

Although health students were able to enter significantly more prescriptions within 15 min using RoboGen2, analysis of the errors made did not show a significant difference between the two versions. This could be due to the heterogeneity in participants’ education background and expertise levels with 52.5% of participants self-identifying as moderate to very experienced in data entry. Furthermore, 30% of the sample population were third-year pharmacy students who are trained from their second year in prescription regulation and data entry. It is not unreasonable to expect that those students with more familiarity with medication names (trade and generic), conversion rates, and prescription entry requirements would produce fewer errors regardless of the software system.

As suggested by Tractinsky (1997), altering the aesthetics of RoboGen2 increased both perceived usability and preference. In this study, while many participants found RoboGen2 to have superior usability, predominantly due to its modernized interface and autofill options, this only worked well for single active medications. Best practice guidelines in NZ promote generic prescribing, and RoboGen2 was designed with this in mind. In some instances, however, prescribers may prefer to prescribe by trade name, for example where different brands have specific formulation characteristics or products with multiple ingredients. This raises a software handling mapping issue between generic and trade names and is a major limitation of the RoboGen2 design.

Of the 20% of health sciences participants who preferred the old system, 62.5% were pharmacy students. Reasons for this preference were the ability to input more medicines and patient information giving users more autonomy in developing their own drug banks. Similarly, some participants preferred manual entry as a safety feature to prevent them from potentially selecting an incorrect formulation from the dropdown menu and jeopardizing patient safety. Medical error can occur at point-of-care where patients are given incorrect medication, wrong dose, or medication at the wrong time (Kushniruk and Borycki, 2008). Although health information technologies (HIT) have been developed to prevent medical error, and improve safety (Kushniruk et al., 2005), they can facilitate error if they fail to take human capability and cognitive limits into account (Kushniruk and Borycki, 2008). Poorly designed systems that contribute to technology-induced errors are strongly related to user interface design (Kushniruk et al., 2005).

A chief aim in HIT development is to match the user’s competencies (Johnson et al., 2005). If a system neglects to meet work demands and workflow practices and is slow and difficult to comprehend, this too can contribute to technology-induced error (Tractinsky, 1997). In this study, pharmacists were accustomed to using HIT in practice and found RoboGen2 easy to use, but the system usability scale was below 68, signifying more work needs to be done to improve usability. Amongst allied health professionals, pharmacists are the highest users of technology in their daily practice, which is thought to be associated with their aptitude for technology use (MacLure and Stewart, 2016). Nonetheless, for Robogen2 to be successfully implemented in pharmacy practice, it needs to be integrated with pharmacists’ current dispensing software systems to keep patient information up to date and accurate. Traditional dispensing systems include clinical decision support systems, such as drug interaction checking, which reduce the risk for medical error and improve quality of care and patient safety (Alotaibi and Federico, 2017), and must be integrated with medication entry software. Integration with dispensing systems also allows pharmacists to connect with medicines formularies and essential drug information resources, as well as medicine prices, subsidized medicines, refill authorizations, medicines reconciliation, inventory management, and more (Odukoya and Chui, 2012). Failure to integrate systems leads to an increase in prescription processing time and has major effects on workflow and load. However, integration only works if there is a standardized set of interfaces between systems, otherwise it requires re-implementation for each pharmacist system. An alternate approach would be to utilize data from a central database of prescriptions. While NZ does have a central database, this store is not available for use outside of general practice and pharmacist systems.

Pharmacists found RoboGen2 more time-consuming to use than their current dispensing software, and the number of tabs awkward. Pharmacists deemed it essential to have patient, prescriber and medication details visible to reduce the risk of duplication or omission of medicines. For medication entry software to be useful with processing repeat prescriptions, it must also be able to recall previous entries to reduce time spent on the activity. Mixed findings have been reported regarding the adoption of HIT in pharmacy to reduce time spent on medication entry. Where some technologies have reduced dispensing time (Motulsky et al., 2008; Rahimi and Timpka, 2011), others were found impractical to use and increased workload (Nanji et al., 2009). As pharmacists may use HIT more comprehensively than other healthcare professionals resulting in additional needs, failing to involve them in HIT testing may result in reduced efficacy and efficiency of the system that could impact patient safety and cause frustration for pharmacists (Darby et al., 2019).

The study had several limitations. Student participants varied widely in age, education, data entry experience, cultural background, and skill level. As participants were health sciences students, results may not generalize to other groups. Recruitment of pharmacists was limited to the central urban Auckland area, the majority of whom were under the age of 30. Whilst NZ community pharmacists use one of two medication dispensing software, pharmacists working in hospitals or general practices may have experiences with software not accounted for in this study. Selection bias could have recruited pharmacists who were more open-minded or interested in technologies, and several pharmacists were recruited from the same pharmacy, increasing the risk for groupthink and perhaps limiting variability of responses. Furthermore, the user will not always be a pharmacist. In the previous COPD study and retirement home study, health professionals who entered the patient medication and other information, were physiotherapists, doctors, or nurses. These healthcare professionals may have different levels of experience and different opinions, and this may mean the robot needs to be tailored to the users.

This paper presents ongoing developments and testing of medication management software for robotic systems. The overall aim of the research was to identify how to improve the usability of the Robogen software for health professionals. The results showed that Robogen2 improved system usability and efficiency but not accuracy, which partially supports the hypothesis. Further developments are necessary to make the software more compatible with existing pharmaceutical systems for pharmacists in particular. Key learnings are that adding safety features and better aesthetics can improve the overall usability and safety of a medication prescription system. By streamlining the workflow and pre-populating data, it is possible to increase the rate that prescriptions are entered, without compromising patient safety. However, the next step could be to integrate the pre-existing pharmacy dispensing systems with specialist medication management systems. This could reduce the overall workload for pharmacists, while incorporating many of the safety features that are built into dispensing systems. Future development of the system for pharmacists should link the software to existing pharmacy systems, and further improve the interface.

## Data Availability

The raw data supporting the conclusion of this article will be made available by the authors, without undue reservation.
